# Time Trends and Prognostic Factors for Overall Survival in Myxoid Liposarcomas: A Population-Based Study

**DOI:** 10.1155/2020/2437850

**Published:** 2020-09-22

**Authors:** Jules Lansu, Winan J. Van Houdt, Michael Schaapveld, Iris Walraven, Michiel A. J. Van de Sande, Vincent K. Y. Ho, Rick L. Haas

**Affiliations:** ^1^Department of Radiation Oncology, Netherlands Cancer Institute, Amsterdam, Netherlands; ^2^Department of Surgical Oncology, Netherlands Cancer Institute, Amsterdam, Netherlands; ^3^Department of Epidemiology, Netherlands Cancer Institute, Amsterdam, Netherlands; ^4^Department of Orthopedic Surgery, Leiden University Medical Center, Leiden, Netherlands; ^5^Department of Research, Netherlands Comprehensive Cancer Organisation (IKNL), Utrecht, Netherlands; ^6^Department of Radiation Oncology, Leiden University Medical Center, Leiden, Netherlands

## Abstract

**Background:**

The purpose of this study was to evaluate the overall survival (OS) and associated characteristics for patients with Myxoid Liposarcoma (MLS) over time in The Netherlands.

**Methods:**

A population-based study was performed of patients with primary localized (*n* = 851) and metastatic (*n* = 50) MLS diagnosed in The Netherlands between 1989 and 2016, based on data from the National Cancer Registry.

**Results:**

The median age of the MLS patients was 49 years, and approximately two-thirds was located in the lower limb. An association was revealed between age and the risk of having a Round Cell (RC) tumor. OS rates for primary localized MLS were 93%, 83%, 78%, and 66% after 1, 3, 5, and 10 years, respectively. The median OS for patients with metastatic disease at diagnosis was 10 months. Increasing age (Hazard Ratio (HR) 1.05, *p*=0.00), a tumor size >5 cm (HR 2.18; *p*=0.00), and tumor location (trunk HR 1.29; *p*=0.09, upper limb HR 0.83; *p*=0.55, and “other” locations HR 2.73; *p*=0.00, as compared to lower limb) were independent prognostic factors for OS. The percentage of patients treated with radiotherapy (RT) increased over time, and preoperative RT gradually replaced postoperative RT. In contrast to patients with localized disease, significant improvement of OS was observed in patients with metastatic disease over time.

**Conclusions:**

In this large nationwide cohort, tumor size and tumor location were independent prognostic factors for OS. Furthermore, a higher probability of an RC tumor with increasing age was suggested. An increased use of RT over the years did not translate into improved OS for localized MLS.

## 1. Introduction

Soft Tissue Sarcomas (STSs) are a heterogeneous group of malignant tumors of mesenchymal origin; therefore, emphasis should be given to characteristics of the various subtypes of STSs in order to adapt treatment strategies to their biological and clinical behavior. Liposarcomas, characterized by adipocytic differentiation, represent one of the most commonly encountered types of STSs. Four histologic subtypes of liposarcoma are recognized: well-differentiated liposarcoma, dedifferentiated liposarcoma, pleomorphic liposarcoma, and myxoid liposarcoma (MLS) [1]. Even among these subtypes, substantial differences exist regarding histology, cytogenetics, clinical behavior, treatment response, and prognosis. MLS, the focus of this study, represents approximately one-third [2] of all liposarcomas and 10% of all adult STSs [3]. MLS generally presents as a slow-growing deep-seated tumor with a predilection for the lower extremity, particularly the groin region [4]. The peak incidence is the fourth and fifth decades of life, which is notably younger than for other liposarcoma subtypes [4]. Distant metastases occur in 14–33% of the patients with MLS [5–13]. In contrast to other STSs, they are characterized by a tendency to metastasize to other soft tissue sites such as the extremities, trunk, retroperitoneum, pleura, pericardium, and even to the bone [2, 14–16]. MLS is relatively sensitive to radiotherapy (RT) [12, 17, 18] and chemotherapy [19, 20], in comparison to other STSs. Local control is achieved in 60–98% of patients [4, 7, 8, 11–14, 18, 21–23], resulting in favorable 5-year overall survival (OS) rates of 67–90% [5, 9, 11–13, 21, 23–25].Although sample sizes in most of the previous studies are small due to the rarity of the disease, several prognostic factors in MLS have been identified. Age at diagnosis >45years, tumor size >10 cm, presence of >5% round cell component, and positive resection margins are associated with worse (disease-specific) survival [5, 7, 8, 11, 13, 21, 23–25]. To overcome these small sample sizes and generate sufficient statistical power, national cancer registries have great potential to come to more definitive conclusions based on unselected observations [24].The purpose of this study was to characterize demographics, with the round cell component in particular (I), OS rates (II) and prognostic factors (III) for MLS in a nationwide retrospective cohort of 901 patients treated between 1989 and 2016 in The Netherlands. Furthermore, time trends of treatment and OS of MLS were studied (IV).

## 2. Patients and Methods

### 2.1. Data Collection

The data were derived from The Netherlands Cancer Registry (NCR). The NCR used the Dutch Pathology Network (PALGA), supplemented with a linkage with the National Registry of Hospital Discharge Diagnosis to identify patients with histologically diagnosed MLS in The Netherlands between 1989 and 2016. Demographics, tumor characteristics, and treatment information were obtained from hospital records by trained NCR registry administrators. The date of death was extracted from the Municipal Personal Records Database. Tumor size was extracted from the *T*-stage in the pathological report, and if not applicable or in case of neoadjuvant treatment, the clinical *T*-stage was used. Reported primary tumor locations were classified to the upper limb, lower limb, trunk, or “other locations”. Other locations included the head and neck, peritoneum, retroperitoneum, mediastinum, and both male and female genital organs. Disease was considered to be primary localized in case of lacking information regarding the metastatic spread, given the very low proportion of cases with primary metastatic disease versus primary localized disease [24] and assuming that a detected distant metastasis at diagnosis is very unlikely not to be reported. A high-grade tumor, from here, termed Round Cell (RC) tumor, was defined as a tumor containing > 5% of Round Cell Component (RCC) [6, 7]. RCC was assessed as a prognostic factor for OS, dichotomized to ≤5% or >5% RCC. From all 903 identified histologically confirmed MLS patients in the NCR database, two patients were excluded because of emigration-related lost to follow-up, and a total of 901 patients were included within this study.

### 2.2. Statistical Analysis

Baseline characteristics are presented as percentage, mean (±standard deviation), or median (+interquartile range (IQR)) in case of a skewed distribution. Differences in baseline characteristics and in patients with localized versus metastatic disease and MLSs versus RC tumors were tested using Student's *t*-tests (continuous variables) and Chi-square tests (categorical variables). The follow-up duration for all patients was calculated as the time between the date of diagnosis and date of death or date of most recent linkage with The Netherlands Population Registry at January 31^st^ 2017. Follow-up for patients alive was estimated using the reverse Kaplan–Meier approach. Patients with primary metastatic disease were analysed separately. OS was analysed by the Kaplan–Meier method, and subgroups were compared by Log-Rank tests. To investigate prognostic factors for OS, aiming to support doctors in informing their patients about their prognosis in the consultation room, cox proportional hazard analyses were performed. A multivariable model was constructed using a backward selection procedure, including variables with *p* values <0.10 [26]. Treatment-related factors were excluded from the model to prevent indication-to-treat bias. A Hazard Ratio (HR) is always presented with a 95% Confidence Interval (CI).Sensitivity analyses were performed to investigate the independent association of RC tumors. For this, we first constructed a univariate model. Then, one potential explanatory variable was added, and the percentual change (∆%) of the HR of the RC tumor variable was calculated by performing an association cox regression model. The localized disease cohort was split into four groups based on their year of diagnosis (quartiles), in order to analyse time trends by mutually comparing those quartiles. Given the relatively small number of primary metastatic cases and, moreover, characterized by a heterogeneity of sites and number of metastases, the metastatic cohort was split into two subgroups (before and after the median year of diagnosis) for time trend analysis, and no prognostic factor analysis was performed. All tests were two-sided, and *p* ≤ 0.05 was considered as statistically significant. Statistical analysis of the data was performed by using IBM SPSS Statistics V25.

## 3. Results

### 3.1. Demographics

**Figure 1:**
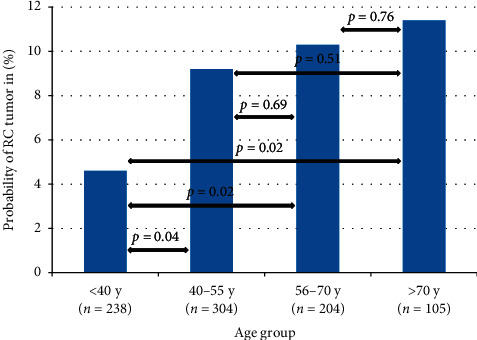
The frequency/proportion of tumors with a RC tumor per age group in localized disease. Overall *p* value by the Chi-square test *p*=0.08.

In the study population, which consisted of 512 (57%) males and 389 (43%) females, the median age at diagnosis was 49 years (IQR 38–62 years). Evidence of distant metastasis at diagnosis was present in 50 (6%) patients. An overview of baseline characteristics is presented in Table 1. In this study population, 77 (9%) of tumors were RC tumors. Patients with RC tumors were significantly older as compared to patients with MLS (median age 53 vs. 48 years, respectively; *p*=0.01) (Supplementary Materials Table 1). The distribution of the probability of having a RC tumor by the age group is shown in Figure 1.

### 3.2. Overall Survival (OS)

The median follow-up for all patients was 7.6 years (IQR 2.4–15.3 years), 8.3 years (IQR 2.8–15.8 years) for patients with localized and 0.8 years (IQR 0.5–1.9 years) for patients with metastatic disease at diagnosis. Median follow-up for patients alive at time of the last follow-up was 13.9 years for localized and 5.9 years for metastatic disease. For all patients with localized disease, the 1-, 3-, 5-, and 10- year OS rates were 93%, 83%, 78%, and 66%, respectively. The OS rates for localized disease, stratified for age, tumor size, RCC, and tumor location are provided in Supplementary Materials Table 2. For patients with metastatic disease at diagnosis, OS rates were significantly lower as compared to localized disease (HR 8.71; 95% CI 6.18–12.28; *p*=0.00). OS rates for metastatic disease after 1, 2, and 3 years were 47%, 29%, and 24%, respectively.

### 3.3. Prognostic Factors in Localized Disease

Increasing age (HR 1.05; 95% CI 1.05–1.06; *p*=0.00), a tumor size > 5 cm (HR 2.42; 95% CI 1.73–3.37; *p*=0.00), RC tumor (HR 1.66; 95% CI 1.20–2.31; *p*=0.00), male gender (HR 1.25; 95% 1.00–1.56; *p*=0.04), and tumor location (trunk HR 1.74; 95% CI 1.34–2.26; *p*=0.00, upper limb HR 0.79; 95% CI 0.47–1.34; *p*=0.38, and other HR 3.78; 95% CI 2.82–5.06; *p*=0.00) with the lower limb as the reference location were found to be significant prognostic factors for OS in univariate analysis. Of note, the lower limb was chosen as the reference location, as this represented the largest subgroup. A positive resection margin (HR 0.92; 95% CI 0.57–1.51; *p*=0.75) did not turn out to be prognostic for OS in univariate analysis. In multivariate analysis, increasing age (HR 1.05; 95% CI 1.04–1.06; *p*=0.00), a tumor size > 5 cm (HR 2.24; 95% CI 1.59–3.16; *p*=0.00), and tumor location (trunk HR 1.29; 95% CI 0.96–1.72, upper limb HR 0.83; 95% CI 0.46–1.52, and other HR 2.73; 95% CI 1.93–3.87, overall *p* value *p*=0.00) remained significant prognostic factors for OS, while RC tumor (HR 1.39; 95% CI 0.97–1.98; *p*=0.07) and male gender (HR 1.24; 95% CI 0.97–1,58, *p*=0.09) did not reach statistical significance. Age and tumor size both explained the association between RC tumors and OS in univariate analysis. After correction for both, the association was attenuated and lost statistical significance (HR 1.36; 95% CI 0.95–1.93; *p*=0.09) (Supplementary Materials Table 3).

### 3.4. Time Trends

**Figure 2:**
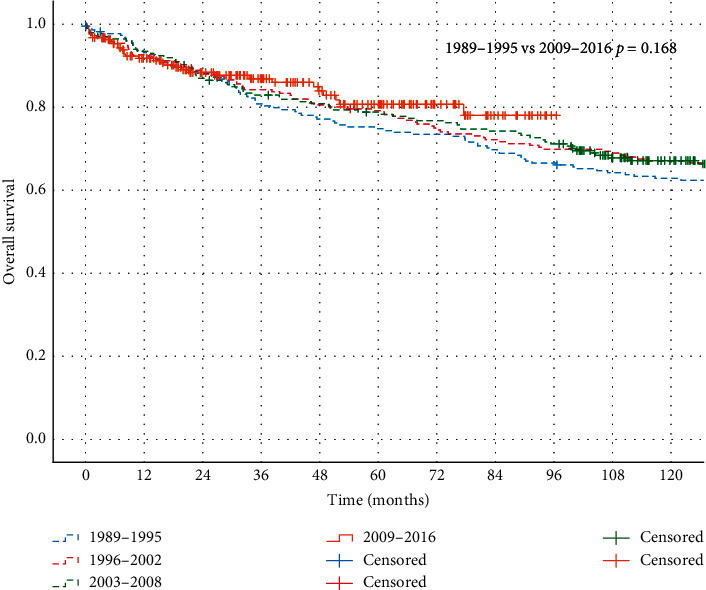
Kaplan–Meier curves representing OS of patients with primary localized disease divided in four groups by the period of diagnoses, as compared by the log-rank test (*p*=0.168).

**Figure 3:**
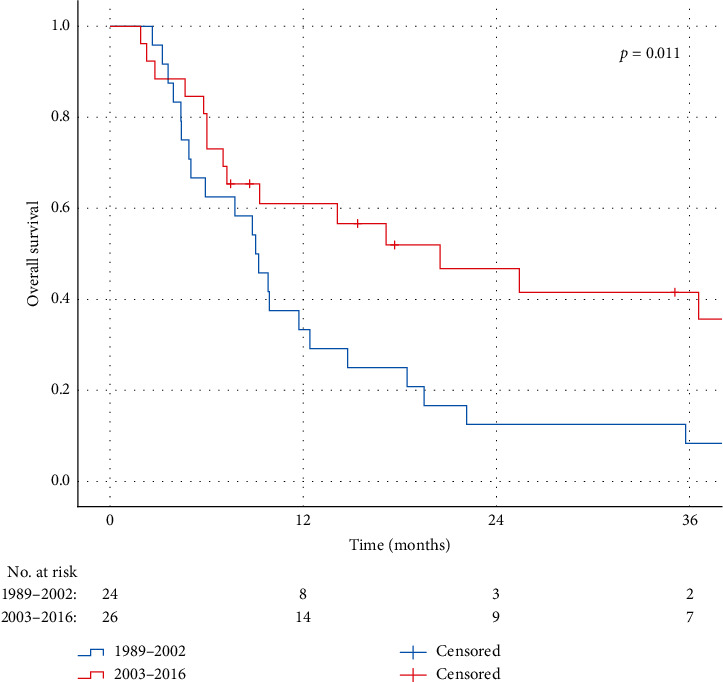
Kaplan–Meier curves representing OS of patients with primary metastasized disease in the period of 1989–2002 vs. 2003–2016, as compared by the log-rank test (*p*=0.011).

**Figure 4:**
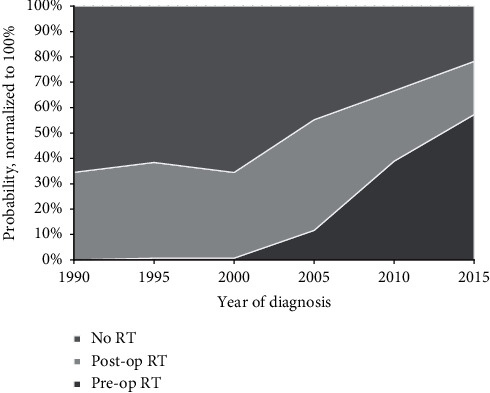
The use and the timing of RT during the study period per year in localized disease.

For localized disease, no significant differences in OS were observed between the four follow-up periods, as shown in Figure 2 . OS rates at 1, 3, and 5 years were 93%, 80%, and 75% in the first period (1989–1995) versus 92%, 87%, and 81% in the last period (2009–2016), respectively (*p*=0.17). In contrast to patients with localized disease, significant improvement in OS rates for patients with primary metastatic disease was observed within the time frame of the study. Median OS for primary metastatic disease increased from 9 months in 1989–2002 to 20 months in 2003–2016. Kaplan–Meier curves of patients divided into the two groups by the year of diagnosis are presented in Figure 3. With respect to time trends in RT treatments, an increase in the use of RT was observed during the study period (Figure 4). Where only 38% of the patients in the first period (1989–1995) received RT, and this was 76% in the most recently diagnosed group of patients (2009–2016, *p*=0.00). Furthermore, postoperative RT was gradually replaced by preoperative RT from 2004 onwards, with a preoperative timing of the RT in 23%, 59%, and 83% of the irradiated patients in 2005, 2010, and 2015, respectively.

## 4. Discussion

In this large nationwide cohort of MLS patients, tumor size and location were found to be independent prognostic factors for OS in localized MLSs. Secondly, an association between a higher incidence of RC tumors and increasing age was revealed. Furthermore, we observed an increased application of, particularly preoperative, RT over time. Prognosis of patients with primary metastatic disease has significantly improved over the years; however, we were unable to demonstrate a significant increase of OS rates for patients with localized disease in recent years. This study confirms the well-established peak incidence [4] and the predilection for males and lower extremities in MLS [5, 8, 10, 12, 24, 27]. In previous series, 7–43% of all MLS were classified as round-cell tumors [5, 7–12], with most authors using RCC cut-off points of 5% [7–12], though some others at 25% [5, 7]. In this series, nearly one in ten tumors was a RC tumor (>5% RCC), belonging to the lowest incidences in the literature. The relationship between age and the incidence of RC tumors has not been reported previously, but was suggested in this study cohort, with a higher probability of having a RC tumor for older patients and an increase of approximately 2% per additional year of age. RC tumors are reported to be associated with inferior outcomes as compared to MLS, with an assumed three to four times higher risk of local recurrence [9, 21] and a higher tendency to metastasize [7, 10], leading to decreased (disease-specific) survival rates in several studies [6, 7, 10]. This reported inferior OS for RC tumors was confirmed in the current study. Furthermore, we showed that both tumor size and increasing age explained part, but not the entire association between RC tumors and OS. These findings might reveal an important subgroup of MLS patients with worse OS in an era of personalized care. More research is needed to determine optimal treatment strategies for these elderly patients with larger tumors with >5% RCC. Obviously, age is a significant prognostic factor in our predictive model, as the outcome measure was OS and not cause-specific survival. We have incorporated age in the model to rule out its role as a confounding factor for other prognostic factors. In accordance with previous reports [8, 10, 23, 28], tumor size was confirmed as independent prognostic factor for OS. Unfortunately, the exact tumor size was not available in our database, necessitating us to use the *T*-stage to derive tumor size information. For that reason, we used 5 cm as a cut-off point for tumor size, instead of the 10 cm cut-off used in most other studies [8, 10, 11, 24, 25]. Even though tumor location is generally considered as a prognostic factor in STSs [27], and to the best of our knowledge, it has never been reported as a prognostic factor for OS in MLS to date. Here, an association between tumor location and OS in MLS was observed; in comparison to the lower extremity, tumors at “other” locations, with the majority consisting of retroperitoneal tumors, have significantly worse OS, independent of confounding factors (HR 2.73, *p*=0.00). Potential contributing factors for worse outcome in retroperitoneal tumors include the challenges of local treatment caused by the complex anatomy of the surgical area. Furthermore, according to the results of immunohistochemical and molecular biological analyses by de Vreeze et al. [29], primary retroperitoneal MLS/RC tumor might be a nonexisting disease, suggesting that a retroperitoneal location either is a metastasis or a misclassified well-/dedifferentiated liposarcoma with the presence of focal myxoid-like changes. In conformity with this hypothesis, the supposed localized “other” location group might contain patients with metastasized disease, resulting in worse OS rates. Tumor location in the trunk did not reach statistical significance in multivariate analysis (HR 1.29, *p*=0.09), but had significantly lower OS in univariate analysis and were, on average, 9 years older, as compared to patients with a tumor located in the lower extremity. With a 5-year OS rate of 78.1% for localized disease, OS is comparable to the rates of 67–94% reported in the literature [5, 9, 11–13, 21, 24]. Prognosis of metastatic disease did improve significantly during the study period, within the last decade in particular. Since 2003 (median year of diagnosis), approximately 40% of patients with metastatic disease at diagnosis were alive 3 years after diagnosis, as compared to 8% of this cohort's patients diagnosed before 2003 and 22% in the study reported by Hoffman et al. (inclusion in the period of 1990–2010) [30]. Advances in imaging techniques, increasing the sensitivity to detect metastases, leading to stage migration, and the introduction of novel systemic therapies (such as trabectedin and eribulin), as well as more aggressive local treatment for metastases including RT may have impacted this improvement [31, 32]. Nevertheless, although consistent with previous reports [24, 28, 33], these diagnostic and therapeutic advances did not translate into significantly improved OS for patients with primary localized disease in recent years. As most of the previously described advances merely affect patients with metastatic disease, while approximately two out of three patients will eventually not metastasize [5, 7, 8, 10, 11, 13, 18], relative gains of these advances are smaller in comparison to the primary metastatic disease cohort. On the other hand, the potential of OS improvement by introduction of effective systemic treatments could possibly have been hampered by the small proportion of patients with localized MLS receiving chemotherapy (6%) as part of their primary treatment. When looking to the Kaplan–Meier curves for localized disease in detail, one can argue that a trend for improved OS is seen in recent years, which could possibly reach statistical significance with longer follow-up. The use of RT increased and its timing to surgery has changed, which is in line with the time trends reported by Lazarev et al. [34]. Where RT was applied strictly in the postoperative setting during the first years of the study period, the use of preoperative RT in The Netherlands commenced in 2004 and from 2010 it even is used more frequently than postoperative RT. The most important limitation of this population-based analysis is the restriction to the variables and outcomes registered by the NCR. For this reason, it was unable to provide local tumor control, metastasis-free-survival, and disease-specific survival as an endpoint in our study. Furthermore, there are some uncertainties regarding the pathology diagnoses. Although data with respect to the translocation status is lacking, MLS diagnoses generally have been translocation confirmed in the last decade of the study period; however, this would not have been the case in earlier years, when molecular diagnostics were not widely available for clinical practice yet. This implicates that the spectrum of myxoid and RC tumors could have changed over the years. Moreover, no central pathology review was performed, although it is custom in The Netherlands to send cases to monthly regional sarcoma pathology board meetings. Lastly, it is important to be aware that recurrences are possible after our median follow-up period of 7.6 years, as the latest reported recurrence occurred at 151 months [9].

## 5. Conclusions

This large nationwide study showed that OS is independently affected by the tumor size and tumor location in MLSs. Furthermore, a higher probability of a RC tumor with increasing age was suggested. We observed an increase in the use of RT in The Netherlands, with a shift from postoperative to preoperative timing in the most recent decade. Nevertheless, OS rates of primary localized MLS remained stable over time. In contrast to primary localized MLS, prognosis of metastatic disease has significantly improved over the years.

## Figures and Tables

**Table 1:** Baseline characteristics of patients with localized and metastatic disease at diagnosis.

Characteristics	Localized disease	Metastatic disease	Total	*p* value
Total patients (*n* = )	851	50	901	
Median follow-up (years)(IQR)	8.3 [2.8–15.8]	0.8 [0.5–1.9]	7.6 [2.4–15.3]	**0.000**
*Gender*				N.S
Male	480 (56%)	32 (64%)	512 (57%)	
Female	371 (44%)	18 (36%)	389 (43%)	

*Tumor location*				N.S.
Upper limb	51 (6%)	2 (4%)	53 (6%)	
Lower limb	551 (65%)	26 (52%)	577 (64%)	
Trunk	170 (20%)	15 (30%)	185 (21%)	
Other	79 (9%)	7 (14%)	86 (9%)	

*Tumor grade*				N.S.
Intermediate grade (MLS)	779 (92%)	45 (90%)	824 (91%)	
High grade (RC tumor)	72 (8%)	5 (10%)	77 (9%)	

*Age (years)*				**0.004**
<40	238 (28%)	8 (16%)	352 (39%)	
40–55	304 (36%)	16 (32%)	312 (35%)	
56–70	204 (25%)	16 (32%)	169 (19%)	
>70	105 (12%)	10 (20%)	68 (7%)	

*TNM classification*				**0.000**
pT0	12 (1%)	1 (2%)	13 (1%)	
pT1	173 (20%)	0	173 (19%)	
pT2	502 (59%)	18 (36%)	520 (58%)	
pTx	164 (19%)	31 (62%)	195 (22%)	
cN0	569 (67%)	21 (42%)	590 (66%)	
cN1	2 (0%)	5 (10%)	7 (1%)	
cNx	280 (33%)	24 (48%)	304 (34%)	
cM0	648 (76%)	0	648 (72%)	
cM1	0	50 (100%)	50 (6%)	
cMx	203 (24%)	0	203 (23%)	

*Surgical resection*				**0.000**
Radical (R0)	368 (43%)	4 (8%)	372 (41%)	
Microscopic irradical(R1)	57 (7%)	2 (4%)	59 (7%)	
Macroscopic irradical (R2)	15 (2%)	7 (14%)	22 (2%)	
Unknown status (Rx)	366 (43%)	8 (15%)	374 (42%)	
No surgery	45 (5%)	29 (58%)	74 (8%)	

*Other treatments*				**0.000**
Radiotherapy (RT)	452 (53%)	14 (28%)	466 (52%)	
Chemotherapy	50 (6%)	19 (38%)	69 (8%)	

The *P* value represents outcome of Chi-square statistical testing (or the Student's *t*-test in case of an continuous variable). Abbreviations: pT = pathological *T*-stage, cN = clinical nodal stage, cM = clinical metastasis stage, N.S. = not significant.

## Data Availability

The data used to support the findings of this study are available from the corresponding author upon request.

## Additional Points

Increased age is associated with a higher probability of having a round-cell component in MLS. The tumor size and tumor location are independent prognostic factors for survival in MLS. The use of, particularly preoperative, radiotherapy for MLS has increased. The overall survival of localized MLS remained stable between 1989 and 2016. The overall survival of metastatic MLS increased between 1989 and 2016.

## Supplementary Materials

Supplementary Materials In this section, the baseline characteristics stratified by the Round Cell Component (RCC) (Table 1) and absolute overall (OS) survival rates stratified by age, tumor size, RCC, and tumor location (Table 2) are presented for patients with localized disease. Furthermore, an overview is given of the associations for OS between RCC and patient and tumor characteristics (Table 3).

## References

[1] Dei Tos A. P. (2000). Liposarcoma: new entities and evolving concepts. *Annals of Diagnostic Pathology*.

[2] Enzinger F. M., Weiss S. M., Enzinger F. M., Weiss S. M. (2001). Liposarcoma. *Soft Tissue Tumors*.

[3] Allen P. W. (1980). Myxoid tumors of soft tissues. *Pathology Annual*.

[4] Bartlett J., Shaaban A., Schmitt F. (2015). Molecular pathology. *Soft Tissue, Bone and Skin Tumors*.

[5] Kilpatrick S. E., Doyon J., Choong P. F. M., Sim F. H., Nascimento A. G. (1996). The clinicopathologic spectrum of myxoid and round cell liposarcoma: a study of 95 cases. *Cancer*.

[6] Smith T. A., Easley K. A., Goldblum J. R. (1996). Myxoid/round cell liposarcoma of the extremities. *The American Journal of Surgical Pathology*.

[7] Antonescu C. R., Tschernyavsky S. J., Decuseara R. (2001). Prognostic impact of P53 status, TLS-CHOP fusion transcript structure, and histological grade in myxoid liposarcoma: a molecular and clinicopathologic study of 82 cases. *Clinical Cancer Research: An Official Journal of the American Association for Cancer Research*.

[8] Fiore M., Grosso F., Lo Vullo S. (2007). Myxoid/round cell and pleomorphic liposarcomas. *Cancer*.

[9] Haniball J., Sumathi V. P., Kindblom L. G. (2011). Prognostic factors and metastatic patterns in primary myxoid/round-cell liposarcoma. *Sarcoma*.

[10] Moreau L.-C., Turcotte R., Turcotte R. (2012). Myxoid\round cell liposarcoma (MRCLS) revisited: an analysis of 418 primarily managed cases. *Annals of Surgical Oncology*.

[11] Ten Heuvel S. E., Hoekstra H. J., Van Ginkel R. J., Bastiaannet E., Suurmeijer A. J. H. (2007). Clinicopathologic prognostic factors in myxoid liposarcoma: a retrospective study of 49 patients with long-term follow-up. *Annals of Surgical Oncology*.

[12] Chung P. W. M., Deheshi B. M., Ferguson P. C. (2009). Radiosensitivity translates into excellent local control in extremity myxoid liposarcoma. *Cancer*.

[13] Nishida Y., Tsukushi S., Nakashima H., Ishiguro N. (2010). Clinicopathologic prognostic factors of pure myxoid liposarcoma of the extremities and trunk wall. *Clinical Orthopaedics and Related Research*.

[14] Cheng E. Y., Springfield D. S., Mankin H. J. (1995). Frequent incidence of extrapulmonary sites of initial metastasis in patients with liposarcoma. *Cancer*.

[15] Pearlstone D. B., Pisters P. W. T., Bold R. J. (1999). Patterns of recurrence in extremity liposarcoma. *Cancer*.

[16] Estourgie S. H., Nielsen G. P., Ott M. J. (2002). Metastatic patterns of extremity myxoid liposarcoma and their outcome. *Journal of Surgical Oncology*.

[17] Pitson G., Robinson P., Wilke D. (2004). Radiation response: an additional unique signature of myxoid liposarcoma. *International Journal of Radiation Oncology∗Biology∗Physics*.

[18] Engström K., Bergh P., Cederlund C.-G. (2007). Irradiation of myxoid/round cell liposarcoma induces volume reduction and lipoma-like morphology. *Acta Oncologica*.

[19] Guadagnolo B. A., Zagars G. K., Ballo M. T. (2008). Excellent local control rates and distinctive patterns of failure in myxoid liposarcoma treated with conservation surgery and radiotherapy. *International Journal of Radiation Oncology∗Biology∗Physics*.

[20] Grosso F., Jones R. L., Demetri G. D. (2007). Efficacy of trabectedin (ecteinascidin-743) in advanced pretreated myxoid liposarcomas: a retrospective study. *The Lancet Oncology*.

[21] Lemeur M., Mattei J.-C., Souteyrand P., Chagnaud C., Curvale G., Rochwerger A. (2015). Prognostic factors for the recurrence of myxoid liposarcoma: 20 cases with up to 8 years follow-up. *Orthopaedics & Traumatology: Surgery & Research*.

[22] Baxter K. J., Govsyeyev N., Namm J. P., Gonzalez R. J., Roggin K. K., Cardona K. (2014). Is multimodality therapy necessary for the management of pure myxoid liposarcomas? a multi-institutional series of pure myxoid liposarcomas of the extremities and torso. *Journal of Surgical Oncology*.

[23] Chowdhry V., Goldberg S., Delaney T. F. (2018). Myxoid liposarcoma: treatment outcomes from chemotherapy and radiation therapy. *Sarcoma*.

[24] Wu J., Qian S., Libin J. (2019). Prognostic factors of patients with extremity myxoid liposarcomas after surgery. *Journal of Orthopaedic Surgery and Research*.

[25] Muratori F., Bettini L., Frenos F. (2018). Myxoid liposarcoma: prognostic factors and metastatic pattern in a series of 148 patients treated at a single institution. *International Journal of Surgical Oncology*.

[26] Halinski R. S., Feldt L. S. (1970). The selection of variables in multiple regression analysis. *Journal of Educational Measurement*.

[27] Pisters P. W., Leung D. H., Woodruff J., Shi W., Brennan M. F. (1996). Analysis of prognostic factors in 1,041 patients with localized soft tissue sarcomas of the extremities. *Journal of Clinical Oncology*.

[28] Kollár A., Rothermundt C., Klenke F. (2019). Incidence, mortality, and survival trends of soft tissue and bone sarcoma in Switzerland between 1996 and 2015. *Cancer Epidemiology*.

[29] de Vreeze R. S., de Jong D., Tielen I. H. (2009). Primary retroperitoneal myxoid/round cell liposarcoma is a nonexisting disease: an immunohistochemical and molecular biological analysis. *Modern Pathology*.

[30] Hoffman A., Ghadimi M., Demicco E. (2012). Localized and metastatic myxoid/round cell liposarcoma. *Cancer*.

[31] Ratan R., Patel S. R. (2017). Trabectedin and eribulin: where do they fit in the management of soft tissue sarcoma?. *Current Treatment Options in Oncology*.

[32] Saponara M., Stacchiotti S., Gronchi A. (2017). Pharmacological therapies for liposarcoma. *Expert Review of Clinical Pharmacology*.

[33] Vos M., Blaauwgeers H. G. T., Ho V. K. Y. (2019). Increased survival of non low-grade and deep-seated soft tissue sarcoma after surgical management in high-volume hospitals: a nationwide study from The Netherlands. *European Journal of Cancer*.

[34] Lazarev S., McGee H., Moshier E. (2017). Preoperative vs postoperative radiation therapy in localized soft tissue sarcoma: nationwide patterns of care and trends in utilization. *Practical Radiation Oncology*.

