# Supplementary material for: Time Trends and Prognostic Factors for Overall Survival in Myxoid Liposarcomas: A Population-Based Study
Source: Sarcoma. 2020 Sep 22;2020:2437850. doi: 10.1155/2020/2437850 (PMC7528038; doi:10.1155/2020/2437850)
Supplement: Supplementary Materials — In this section, the baseline characteristics stratified by the Round Cell Component (RCC) (Table 1) and absolute overall (OS) survival rates stratified by age, tumor size, RCC, and tumor location (Table 2) are presented for patients with localized disease. Furthermore, an overview is given of the associations for OS between RCC and patient and tumor characteristics (Table 3). [file 2437850.f1.pdf]

**Supplementary data**

**Table 1.** Baseline characteristics of localized disease stratified by RCC

| Characteristics | MLS (≤ 5% RCC) | RC tumor (> 5% RCC) | Total | p-value |
|---|---|---|---|---|
| Total patients (n=) | 779 | 72 | 851 | |
| *Age (years)* | | | | **0.009** |
| Median age (IQR) | 48 (38-61) | 53 (45-68) | 49 (38-61) | |
| *Tumor size* | | | | 0.438 |
| ≤ 5 cm | 163 (21%) | 12 (17%) | 175 (21%) | |
| > 5cm | 546 (70%) | 52 (72%) | 598 (70%) | |
| *Gender* | | | | 0.923 |
| Male | 439 (56%) | 41(56%) | 480 (56%) | |
| Female | 340 (44%) | 31 (43%) | 371 (44%) | |
| *Tumor location* | | | | 0.263 |
| Upper Limb | 49 (6%) | 2 (3%) | 51 (6%) | |
| Lower Limb | 507 (65%) | 44 (56%) | 551 (64%) | |
| Trunk | 150 (19%) | 20 (28%) | 170 (21%) | |
| Other | 73 (9%) | 6 (8%) | 79 (9%) | |

**Table 2.** Localized myxoid liposarcoma: OS by age, tumor size, RCC and tumor location.

| Absolute OS rates in primary localized disease | | | | |
|---|---|---|---|---|
| | **1 year** | **3 year** | **5 year** | **10 year** |
| ***Age (years)*** | | | | |
| *< 40 (n=238)* | 99% | 95% | 91% | 79% |
| *40-55 (n=304)* | 96% | 89% | 84% | 74% |
| *56-70 (n=204)* | 93% | 78% | 72% | 64% |
| *> 70 (n=105)* | 71% | 54% | 47% | 23% |
| ***Tumor size*** | | | | |
| *≤ 5cm (n=175)* | 98% | 94% | 92% | 85% |
| *> 5cm (n=598)* | 93% | 82% | 76% | 64% |
| ***RCC*** | | | | |
| *MLS (n=779)* | 94% | 84% | 79% | 68% |
| *RC tumor (n=72)* | 85% | 73% | 68% | 52% |
| ***Tumor Location*** | | | | |
| *Lower Limb (n=551)* | 97% | 89% | 84% | 73% |
| *Trunk (n=170)* | 86% | 75% | 70% | 57% |
| *Upper Limb (n=51)* | 98% | 92% | 92% | 81% |
| *Other (n=79)* | 76% | 55% | 47% | 35% |
| ***All patients with localized disease (n=851)*** | 93% | 83% | 78% | 66% |

**Table 3.** Association Cox regression Model to test the association for OS between RCC and patient and tumor characteristics

| Characteristics | HR (95% CI) | P | Δ% |
|---|---|---|---|
| **RC tumor** | 1.66 (1.20-2.31) | 0.002 | |
| **+ Age** | 1.44 (1.04-1.99) | 0.030 | -13 |
| **+ Tumor size >5cm** | 1.55 (1.09-2.21) | 0.015 | -7 |
| **+ Male gender** | 1.67 (1.21-2.32) | 0.002 | 0 |
| **+ Tumor location** | 1.76 (1.26-2.44) | 0.047 | 6 |
| **+ Age & tumor size >5cm** | 1.36 (0.95-1.93) | 0.093 | -18 |
